# A comparative study on the production of extracellular hydrolytic enzymes of *C. albicans* and non-*albicans Candida* species isolated from HIV^+^/AIDS patients and healthy individuals

**DOI:** 10.18502/cmm.8.2.10330

**Published:** 2022-06

**Authors:** Fatemeh Fathi, Ali Zarei Mahmoudabadi, Mahnaz Fatahinia

**Affiliations:** 1 Department of Medical Mycology, School of Medicine, Ahvaz Jundishapur University of Medical Sciences, Ahvaz, Iran; 2 Infectious and Tropical Diseases Research Center, Health Research Institute and Department of Medical Mycology, School of Medicine, Ahvaz Jundishapur University of Medical Sciences, Ahvaz, Iran

**Keywords:** AIDS patients, *Candida* species, Enzyme activity, Oral candidiasis

## Abstract

**Background and Purpose:**

Oropharyngeal candidiasis is the most prevalent opportunistic fungal infection in patients with human immunodeficiency virus (HIV) as well as other immunodeficiency disorders, which is caused by various *Candida* species, mostly *Candida albicans*. Studies have shown that *Candida* isolates differ in their pathogenicity. These variations are attributed to virulence factors, host characteristics, and the target tissue. This study aimed to determine and compare the secretion of hydrolytic enzymes in *C. albicans* and non-*albicans Candida* species isolated from HIV^+^/AIDS patients and healthy individuals.

**Materials and Methods:**

Samples were taken from 201 patients with HIV and 118 healthy individuals. The samples were identified by macroscopic, phenotypic, and molecular methods, and virulence factors were subsequently measured. Statistical differences in enzymatic activity of various *Candida* isolates were calculated (*P*<0.0001).

**Results:**

In total, 95 samples (47.20%) from patients and 46 samples (38.90%) from healthy individuals were positive for the growth of different Candida species. There were 39 (41.10%) and 36 (78.30%) *C. albicans* in patients and healthy individuals, respectively, as well as 56 (58.90%) and 10 (21.70%) non-*albicans* species in patients and healthy subjects, respectively. All the enzymes produced by *Candida* species enzymes were at low, medium, and high levels. Hemolysin activity in *Candida* species isolated from patients was significantly higher, compared to healthy individuals. Moreover, the activity of all *C. albicans* enzymes in patients was significantly higher than other *Candida* species.

**Conclusion:**

The *C. albicans* isolated from HIV-positive individuals secreted higher amounts of exoenzymes, and can cause oropharyngeal candidiasis and become a source of candidiasis for the host.

## Introduction

*Candida* species are one of the most common agents of opportunistic fungal diseases in humans, leading to acute, subacute, or chronic infections of the skin, nails, vaginal mucosa, bronchi, lungs, and gastrointestinal tract [ 1 ].

Oropharyngeal candidiasis is an opportunistic infection of the oral cavity, which is caused by the overgrowth of *Candida* species, and *Candida albicans* is the most common agent in this regard. The severity of the disease varies depending on age and several predisposing factors. Candidiasis can also be a sign of systemic diseases and a frequent problem among immunocompromised individuals [ 2 ]. The oral lesions of *C. albicans* are among the first signs of acquired immune deficiency syndrome (AIDS) in patients infected with human immunodeficiency virus (HIV), indicating disease progression and reduction of CD4^+^ lymphocytes. 

The AIDS is one of the most important diseases of the cellular immune system caused by HIV. The AIDS patient becomes susceptible to opportunistic infectious diseases, like candidiasis, due to the invasion of this virus into CD4^+^ lymphocytes and the destruction of these cells that play a role in commanding the cellular defense system. Approximately 90% of people living with HIV develop oral lesions due to this yeast at least once during the time of their disease [ 3 ].

The researchers have shown that *Candida* isolates are different in their pathogenicity. These differences are attributed to virulence factors and characteristics of the host and the target tissue [ 4 ]. The main virulence factors of *C. albicans* include the production of hydrolytic enzymes (proteinase, phospholipase, hemolysin, and esterase), hyphae formation, biofilm formation, and the expression of drug-resistant genes that are involved in the degradation process of the host cell membrane. These enzymes are also involved in the binding of *Candida species* to the target tissue, especially in the hyphae stage [ 5 , 6 ]. 

Proteinase is secreted during adhesion, colonization, and attack on host tissue, which is responsible for the hydrolysis of albumin, creatine, collagen, hemoglobin, immunoglobulins, and extracellular matrix proteins [ 7 ]. In particular, hydrolytic enzymes, such as phospholipase, esterase, and hemolysin are more important as they are implicated in adhesion, attack, and nutrient uptake for the fungus while disrupting host immune mechanisms [ 8 ]. Studies have shown that *C. albicans* isolates taken from different clinical specimens produce one or more of these factors depending on the site of isolation as well as the clinical condition, immune status, and treatment regimen of the patient [ 9 ].

Given that the use of antiviral drugs, such as highly active antiviral therapy (HAART), decreases the incidence and severity of oropharyngeal candidiasis in patients with AIDS, some researchers have suggested that among six drug classes used to treat this group of patients, protease inhibitors may reduce or even inhibit exoenzyme activity of proteinases in *Candida* species [ 10 ]. A study of the relationship between the activity of hydrolytic enzymes in different species of *Candida* and the development of oropharyngeal candidiasis among HIV-positive patients can be an important tool to examine the pathogenesis of this organism in these patients. The results of such studies can be used to adopt appropriate health and treatment strategies to control and prevent the progression of disease in patients [ 11 , 12 ].

This study aimed to determine and compare the activity of hydrolytic enzymes (hemolysin, esterase, phospholipase, and proteinase enzymes) in *C. albicans* and non-*albicans Candida* species isolated from HIV^+^/ADIS patients and healthy individuals.

## Materials and Methods

The present research was confirmed by the Ethics Committee of Ahvaz Jundishapur University of Medical Sciences, Ahvaz, Iran, and registered with IR.AJUMS.MEDICINE.REC.1398.048 code. In this regard, permission was obtained to take samples from patients infected with HIV^+^/AIDS who were referred to Ahvaz Behavioral Diseases Counseling Center as well as from healthy individuals (i.e., control group). The inclusion criterion for patients infected with HIV^+^/AIDS was a definite HIV-positive result. Moreover, the inclusion criterion for the healthy group was the absence of predisposing factors for candidiasis, such as diabetes and suppressive diseases of the immune system, such as cancer. It should be noted that the exclusion criterion was grown specimens with several colonies less than 10 (colony-forming unit/swab<10).

### Sampling

Samples were taken with sterile wet swabs from 201 patients and 118 healthy individuals. Swabs were applied to all parts of the oral mucosa of patients and healthy individuals and subsequently cultured on CHROMagar^TM^ Candida (Paris, France) and incubated at 35 °C for 24-72 h. 

### Identification of isolates by macroscopic and classical methods

The culture medium was evaluated in terms of both colony color and count based on colony-forming unit/swab [ 13 ]. The colonies were subsequently identified based on the morphology of isolates on Corn meal agar medium (Difco, USA), Germ Tube Test, and growth at 45 °C.

### Polymerase chain reaction and restriction fragment length polymorphism test to identify fungal isolates

To identify Candida species, the DNA from each sample was extracted by the boiling method [ 14 , 15 ], and the internal transcribed spacer (ITS) region of ribosomal DNA was amplified using a pair of primers (ITS1 and ITS4) [ 13 ]. After observing the resulting bands and assuring the quality of products using agarose gel electrophoresis, the products were digested by *MspI* restriction enzyme to confirm the species, and each yeast was distinguished according to the electrophoretic pattern of each species. Differentiation of *C. albicans* from *C. dubliniensis* was performed using the duplex polymerase chain reaction (PCR) method (Primers include CALF 5´- TGGTAAGGCGGGATCGCTT-3´, CALR 5´-GGTCAAAGTTTGAAGATATAC-3´, for *C. albicans* and, CDUF 5´-AA ACTTGTCACGAGATTATTTTT-3´, CDUR 5´-AAA GTTTGAAGAATAAAATGGC-3´ for *C. dubliniensis*) [ 13 ]. 

### Measurement of hydrolytic enzymes in vitro

### Preparation of yeast suspension

A yeast suspension with 0.5 McFarland turbidity (0.5-2.5×10^6^ yeast per ml) was prepared from *Candida* isolates using a spectrophotometer (OD=530), and 10 μl of each suspension was inoculated onto plates in duplicate [ 10 ].

### Determination of phospholipase activity

Phospholipase activity was determined using an egg agar medium based on Samaranayake et al. method [ 16 ]. After inoculation of the suspension prepared from yeast isolates into the culture medium, the media were incubated at 37 °C for 1 week, after which they were examined for the presence of a white and milky halo around the colony.

### Determination of esterase activity

Esterase activity was measured using Tween 80 turbidity test medium according to the Slifkin method [ 17 ]. Afterward, the standard suspension of each yeast was inoculated into a culture medium and incubated for 1 week at 29 °C. Afterward, the presence of a clear halo around each colony was examined.

### Determination of proteinase activity

The proteinase production by *Candida* isolates was measured using bovine serum experimental medium. Inoculation of each strain was then performed, which was incubated for 1 week at 37 °C. Proteinase activity was recorded based on the diameter of the transparent halo region [ 10 ].

### Determination of hemolytic activity

Hemolysin production was assessed by the modified Luo et al. method [ 18 ]. Briefly, the standard suspensions obtained from the strains were inoculated into the medium and incubated for 2 days at 37 °C. Hemolysin activity was then measured by determining the clear halo around the colony [ 10 , 19 ].

### Statistical analysis

The data were analyzed using the two-sample t-test and Mann-Whitney test in the SPSS software (version 23). Differences between groups were considered statistically significant at *P*<0.05.

## Results

### Demographic characteristics of the subjects

During this study, samples were taken from 201 HIV-positive patients and 118 healthy individuals. In total, 319 oral samples were cultured, and 95 (47.20%) and 46 (38.90%) samples from infected patients and healthy subjects were positive for the growth of *Candida* species, respectively. Tables 1 and 2 summarize the demographic characteristics of the subjects in terms of age, education level, and the frequency of *Candida* species isolated from patients and healthy individuals. The mean ages of patients and healthy individuals were 40.62 and 39.58 years, respectively. It should be mentioned that the *p*-value was 0.676 and the results were not significant at *p* < 0.05 (the chi-square statistic with Yates correction *p*-value=0.112).

**Table 1:** Demographic characteristics and frequency of species isolated from patients and healthy individuals

Variables	Healthy (N) (%)	HIV^+^ (N) (%)	*P-value*
Age Range (Years)			
2—20	4 (9)	7 (7)	0.505£
21—40	25 (54)	43 (45)	
≥41	17 (37)	45 (48)	
Total	46 (100)	95 (100)	
Gender			
Male	26 (57)	68 (72)	0.075£
Female	20 (43)	27 (28)	
Total	4 (100)	95 (100)	
Education level			
University education	28 (60.87)	2 (2.11)	<0.0001
No university education	18 (39.13)	93 (97.90)	
Total	46 (100)	95 (100)	
*Candida* species*			
*Candida albicans*	36 (78.30)	39 (41.1)	<0.0001
*Candida* non-*albicans*	10 (21.7)	56 (58.9)	
Total	46 (100)	95 (100)	

* Fisher’s exact test with *P<0.0001*

£ The chi-square test with *P<0.05*

**Table 2:** Frequency of different *Candida* species isolated based on molecular methods

*Candida* species	Healthy subjects	HIV-positive patients	Total number
	Number (%)	Number (%)	Number (%)
*C. albicans*	36 (78.3)	39 (41.1)	75 (53.2)
*C. glabrata*	5 (10.9)	14 (14.7)	19 (13.5)
*C. dubliniensis*	3 (6.5)	16 (16.8)	19 (13.5)
*C. parapsilosis*	0 (0)	5 (5.3)	5 (3.5)
*C. tropicalis*	0 (0)	8 (8.4)	8 (5.7)
*C. guilliermondii*	0 (0)	3 (3.2)	3 ( 2.1)
*C. krusei*	1 (2.2)	1 (1.1)	2 (1.4)
*C. kefyr*	1 (2.2)	3 (3.2)	4 (2.8)
*C. spp*	0 (0)	6 (6.3)	6 (4.3)
Total	46 (100)	95 (100)	141 (100)

### Molecular test results

**Figure 1:**
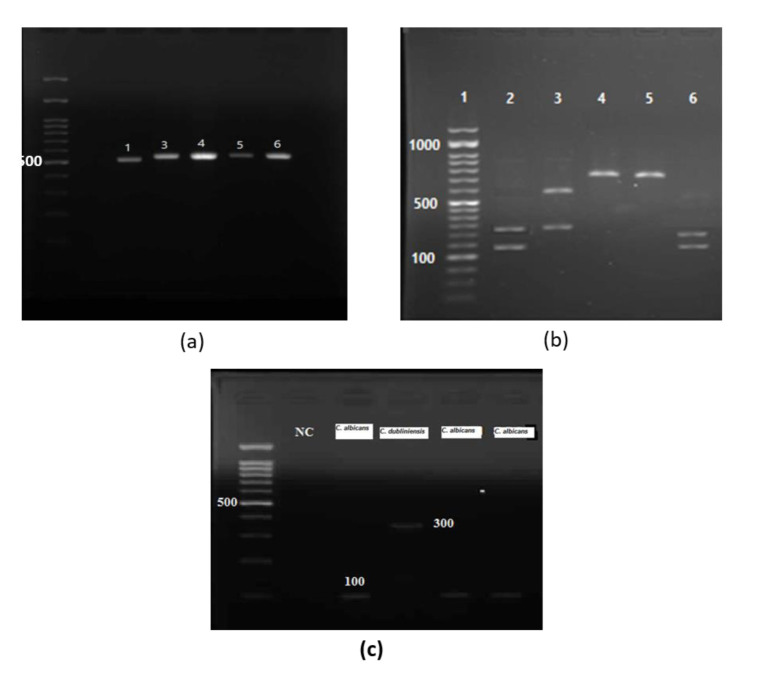
a) Electrophoresis image of PCR products from ITS regions on 1.2% agarose gel related to possible *C. albicans* or *C. dubliniensis* isolates b) Image of PCR-RFLP electrophoresis products of ITS region by MSPI enzyme respectively (1: Marker; 2: *C. tropicalis*; 3: *C. glabrata*; 4 & 5: C. kefyr; 6: *C. albicans*) c) Electrophoresis image of PCR products from Duplex PCR

According to the PCR and restriction fragment length polymorphism (RFLP(-PCR tests of ITS regions, all the isolates taken from patients and healthy individuals were identified and confirmed as different species of *Candida* (*C. albicans*, *C. glabrata*, *C. dubliniensis*, *C. parapsilosis*, *C. tropicalis*, *C. guilliermondii*, *C. krusei*, and *C. kefyr*) (Figure 1).

**Figure 2:**
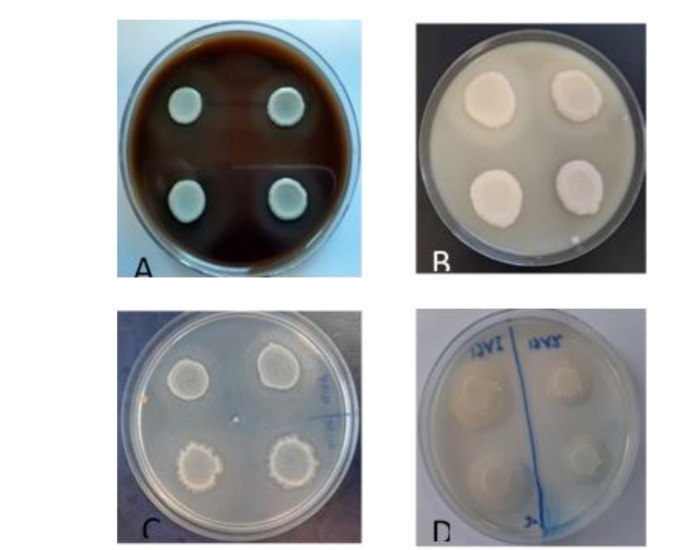
Enzymatic activity of *Candida* species (A): Hemolytic activity as a clear halo; (B): Proteinase activity as clear halo; (C): Esterase activity as star sediment; (D): Phospholipase activity as sediment

A comparison of the activity of esterase, proteinase, phospholipase, and hemolysin enzymes secreted by *Candida* species isolated from the oral cavity of patients and healthy subjects was performed using the Mann-Whitney test, according to which only hemolytic activity showed a significant difference (*P*<0.0001) (Figure 2 and Table 3).

**Table 3:** Enzymatic activity of *Candida* species isolated from oral of patients and healthy subjects

Enzymatic activity(EA)	Health (N) (%)	HIV^+^ (N) (%)	P-value
Esterase			
NP	5 (10.9)	22 (23.20)	
HP	13 (28.30)	19 (20)	
LP	4 (8.70)	17 (17.90)	
IP	24 (52.20)	37 (38.90)	
Total	46 (100)	95 (100)	0.400
Proteinase			
NP	13 (28.30)	22 (23.20)	
HP	2 (4.30)	2 (2.10)	
LP	11 (23.90)	10 (10.50)	
IP	20 (43.50)	61 (64.20)	
Total	46 (100)	95 (100)	0.123
Phospholipase			
NP	7 (15.20)	0 (0.00)	
HP	6 (13.00)	6 (6.30)	
LP	8 (17.40)	12 (12.60)	
IP	25 (54.40)	77 (81.10)	
Total	46 (100)	95 (100)	0.071
Hemolysin			
NP	7 (15.20)	0 (0.00)	
HP	1 (2.20)	40 (42.10)	
LP	2 (4.30)	0 (0.00)	
IP	36 (78.30)	55 (57.90)	
Total	46 (100)	95 (100)	0.0001>

EAI: enzymatic activity Index; HP: high production; 0.39 ≤ EAI ≤ 1; LP: low production; 0.70 ≤ EAI ≤ 0.99; IP: intermediate production 0.40 ≤ EAI ≤ 0.69; NP: Non-production

Statistical indices of activity of each enzyme were separately determined for *C. albicans* and non-albicans *Candida* species in patients and healthy individuals based on a two-sample t-test and Mann-Whitney test. In the healthy group, the esterase enzyme had a significant difference, and in the patient group, phospholipase, esterase, and proteinase enzymes showed a significant difference (Table 4).

**Table 4:** Activity of each enzyme in *Candida albicans* and non-*albicans Candida* species in patients and healthy individuals

Fungus	HIV-positive groups N=95			Healthy group N=46		P¥
	*C. albicans* N=39	Non-albicans N=56	P	*C. albicans* N=36	Non-albicans N=10	
Enzyme	SD Mean	SD Mean		SD Mean	SD€ Mean	
Proteinase	(0.15)0.63	(0.22) 0.69	0.001¥	(0.18) 0.68	(0.17) 0.75	0.182
Hemolysin	(0.11) 0.42	(0.10) 0.42	0.976*	(0.12) 0.54	(0.23) 0.71	0.068
Esterase	(0.20) 0.51	(0.24) 0.72	0.0001¥	(0.19) 0.53	(0.28) 0.86	0.005
Phospholipase	(0.11) 0.52	(0.10) 0.61	*0.0002	(0.19) 0.60	(0.18)0.78	0.014

* Two-sample t-test

¥ Mann-Whitney test

€ Standard Deviation

## Discussion

The incidence of fungal infections, especially *Candida* species, has increased significantly over the past two decades. In human beings, *Candida* is part of the commensal flora and nevertheless causes progressive infections, which has now become the fourth leading factor of nosocomial infections [ 20 ]. The AIDS is one of the most important diseases of the cellular immune system predisposing a person to opportunistic infectious diseases, such as candidiasis [ 21 ]. Studies have shown that the severity of infection in HIV-positive patients is proportional to the host immune deficiency. However, genotypic and phenotypic investigations have revealed a strong relationship between the natural selection of strains and the increasing virulence of colonized *Candida* species in the oral cavity of these individuals [ 22 ].

One of the objectives of this study was to obtain epidemiological information on *Candida* colonization to identify different species of *Candida* isolated from HIV-positive patients in comparison with healthy individuals who live in Ahvaz.

In the present study, the prevalence of *Candida* was 38.90% in healthy subjects, which significantly increased to 47.20% in HIV-positive patients. Although *C. albicans* was the most frequent Candida species in both patients and healthy groups, the prevalence of non-albicans *Candida* species in patients and healthy individuals was 41.10% and 78.30%, respectively, which was significantly different (*P*<0.0001). 

These findings indicate a high growth of the population of non-albicans *Candida* species in those infected with AIDS and possibly an increase in the pathogenicity of these species. In various investigations, *Candida* has been found to be present in the oral cavity of 10-30% of healthy people, the colonization of which appears to increase significantly under certain conditions, including AIDS. Results of the present study are consistent with those of the studies performed in China (49.50%), Brazil (48%), Taiwan (51.40%), and Iran (43.80%) regarding the prevalence of *C. albicans* [ 13 , 20 ]. 

In a study conducted by Heydarian et al. in Isfahan, Iran, the rate of *Candida* colonization in people with AIDS was 68.80%. Moreover, in a study carried out by Katiraee et al. in Tehran, Iran, 77.20% of patients were colonized, which is not consistent with the prevalence of *Candida* in the present study. The sampling in the study performed by Katiraee was performed at Tehran AIDS Research Center affiliated with Imam Khomeini Hospital, which is specifically dedicated to AIDS patients. In the aforementioned study, 59.90% of the patients had apparent symptoms of oral candidiasis, while the participants of the present study were HIV-positive people under antiviral treatment without any symptoms of oral candidiasis who only referred for their periodic medical care. 

The difference between the level of colonization in the study of Heydarian et al. in Isfahan and that in the present research can be due to the variation in climatic conditions of sampling, type of nutrition, and the culture medium since the rate of *Candida* isolation in healthy individuals was also higher than that in the present study. Besides, the number of samples studied in the present research was about three times more than that of the aforementioned study [ 21 , 23 ].

According to the demographic information of the patients with AIDS, a high percentage of patients (97.90%) did not have university education. These findings clearly show the need to increase the awareness and education level of individuals in the community to prevent and reduce the incidence of HIV/AIDS and thereby, the prevalence of *Candida* in their mouths (Table 1). It is noteworthy that other studies have revealed a direct relationship between education and the prevention of HIV infection [ 24 , 25 ]. 

In a study conducted by Bunyasi et al. in South Africa, a negative relationship (OR) was noticed between education level and the prevalence of HIV infection, indicating that the prevalence of AIDS decreases significantly with the increase in years of formal education. These changes and differences depend on the provinces, economic conditions, and individual assets of the subjects [ 26 ]. In Kenya, Mwamwenda et al. studied 257 people from various schools and universities and concluded that education continues to be the most important social vaccine, which can prevent, control, and manage AIDS in the community [ 27 ].

In the present study, through the detection of different species of *Candida* based on morphological and molecular methods in HIV-positive patients, eight different species were identified. Accordingly, the highest frequency belonged to *C. albicans* (41.10%), followed by *C. dubliniensis* (16.80%) and *C. glabrata* (14.70%). Moreover, in healthy individuals, five different species of *Candida* were isolated, and the highest frequency belonged to *C. albicans* (78.30%), followed by *C. glabrata* (10.90%) and *C. dubliniensis* (6.50%). 

As mentioned, the prevalence of non-*albicans Candida* species in AIDS patients had a significant increase, compared to healthy individuals (P<0.0001). The 58% and 90% prevalence of non-*albicans Candida* species in people with AIDS versus 21.70% incidence among healthy individuals indicates an increase of pathogenicity in patients. The emergence of new non-*albicans* species in these individuals is worrying. The results of a study performed by Anwar Khan P et al. were different from those of the present investigation in terms of the frequency percentage of *C. albicans* (60%), which finally identified five different species of *Candida* perhaps due to the absence of molecular methods for the detection of *Candida* species [ 28 ]. However, the results of the present study were more consistent with those of the research performed by Maheshwari et al. and Lahkar et al. in terms of species diversity [ 29 , 30 ].

In a study carried out by Arati Mane et al. in 2012 in India on 335 samples (210 HIV-positive and 125 HIV-negative), it was found that *C. albicans* isolates collected from HIV-positive samples had a significant increase in virulence factors, such as proteinase, phospholipase, and hemolysin as well as the ability to adhere, compared to HIV-negative samples [ 31 ]. In the aforementioned study, only *C. albicans* was investigated similar to the present research, and the activity of all four enzymes related to *C. albicans* increased in the patient group, compared to the control group. 

Based on the results of the present study, all isolates had hemolytic activity in patients, while 15.2% of the isolates lacked hemolytic enzyme activity in healthy individuals (*P*<0.0001). Hemolysin secreted by *Candida* species destroys blood cells and releases iron in them. As a result of the increase in iron and its transfer to the *Candida* yeast environment, the growth of yeasts increases, and thereby, the fungal infection is reinforced [ 10 ]. 

An increase in the secretion of enzymes depends on the physiological conditions of the target tissue. Higher expression of proteinase activity in *Candida* isolates taken from HIV-infected individuals, compared to non-infected ones can be attributed to the lower pH of their mouths due to dryness of the mouth as well as the change in their salivary composition. However, the advent of HAART in 1997 and the effect of these antiviral drugs on *C. albicans* through protease inhibitors have reduced *C. albicans* infections. It should be noted that HIV and *C. albicans* proteases are similar and the production of proteases from strains isolated from those consuming these drugs is also expected to decrease. The results of the present study showed that 23.2% of the isolates from HIV-positive patients lacked proteinase activity [ 7 ].

In this study, 100% and 88.4% of the isolates in patients and healthy individuals were able to produce phospholipase, respectively. *Candida* extracellular phospholipases help in the invasion of host mucosal epithelium. It seems that phospholipase aids in pathogenesis by lysing host cells or altering their surface properties to facilitate adhesion and penetration. Phospholipase production in isolates of HIV-positive individuals was consistent with other reports [ 31 , 32 ]. 

In the present study, 2.23% and 9.10% of the isolates collected from patients and healthy individuals were unable to produce esterase, respectively. Moreover, the mean esterase activity of isolates in patients was higher, compared to that in healthy individuals which were consistent with the study performed by Maheronnaghsh et al. [ 10 ]

## Conclusion

By evaluating the secretion of exoenzymes, especially hemolysin, proteinase, and phospholipase by Candidiasis agents isolated from the mouth, it is possible to find the severity of Candidiasis pathogenicity in HIV-positive patients. Due to the importance of this issue, it is suggested that systematic analysis be performed on factors related to various diseases. According to the results of this study, it was concluded that *C. albicans* isolated from HIV-positive individuals who secrete higher amounts of these enzymes was highly pathogenic and that could cause oropharyngeal candidiasis and be a source of disseminated Candidiasis in the host.

## Acknowledgments

The authors are thankful to the staff of Ahvaz Behavioral Diseases Counseling Center for cooperation in this study. This study was supported by a grant as approved at Ahvaz Jundishapur University of Medical Sciences, Ahvaz, Iran (grant No. OG- 9849).

## Authors’ contribution

M. F. was involved in the study design and interpretation of the data of the study and the final edition of the manuscript. F. F. contributed to all the steps of experimental work, collection, and preparation of clinical samples, data analysis, and preparation of the manuscript draft. A. Z. M. contributed interpretation of the data. 

## Conflicts of interest

The authors declare no conflict of interest.

## Financial disclosure

There are no financial conflicts of interest to disclose.
